# Novel Diagnostic Tool for Detecting Cognitive Impairment by Integrating Functional Near‐infrared Spectroscopy and Plasma Biomarkers in a Large Chinese Cohort

**DOI:** 10.1002/alz.086016

**Published:** 2025-01-09

**Authors:** Bin Jiao, Ziyu Ouyang, Sizhe Zhang, Cong Zhang, Xuewen Xiao, Yiliang Liu, Yuzhang Bei, Yuan Zhu, Qijie Yang, Tianyan Xu, Xiaoli Hao, Xuan Yang, Meidan Wan, Shilin Luo, Beisha Tang, Lu Shen

**Affiliations:** ^1^ Xiangya Hospital, Central South University, Changsha, Hunan China; ^2^ Jili Hospital, Changsha, Hunan China

## Abstract

**Background:**

An objective and effective screening tool for cognitive impairment (CI) is needed in the Chinese population. However, whether the integration of brain resting‐state functional connectivity (rs‐FC) and neurodegenerative plasma biomarkers can distinguish CI from cognitively normal (CN) individuals remains unclear.

**Method:**

A total of 1077 participants from a longitudinal Liuyang community cohort were enrolled. The rs‐FC was collected using functional near‐infrared spectroscopy (fNIRS). Plasma biomarkers, including p‐tau181, p‐tau217, p‐tau231, Aβ40, Aβ42, NfL, and GFAP were detected using single molecule assay method.

**Result:**

The average FC of the CI group was significantly lower than that of the CN group (p < 0.0001). The CI detection model based on the combined altered FCs, achieved an area under the curve (AUC) of 0.785, which showed better diagnostic performance than that of single plasma biomarker with AUC of 0.592‐0.631. However, the integration of altered FC and plasma biomarker presented the best accuracy with AUC of 0.887‐0.933. Totally, 27 FCs were correlated with plasma biomarkers, and among them, the strength of right part of Brodmann's area 6 vs. right part of Brodmann's area 1+2 (BA6R.vs.BA1+2R) was negatively associated with p‐tau231, p‐tau217 and GFAP. Additionally, 13 FCs could mediate the effects of three plasma biomarkers on cognition, including p‐tau217 (n = 3), NfL (n = 10), and GFAP (n = 1).

**Conclusion:**

Rs‐FC based on fNIRS combined with plasma biomarkers is feasible and effective for non‐invasive CI screening.

